# Nonporous Inorganic Nanoparticle-Based Humidity Sensor: Evaluation of Humidity Hysteresis and Response Time

**DOI:** 10.3390/s20143858

**Published:** 2020-07-10

**Authors:** Shinya Kano, Harutaka Mekaru

**Affiliations:** Sensing System Research Center, National Institute of Advanced Industrial Science and Technology (AIST), Tsukuba 305-8564, Japan; h-mekaru@aist.go.jp

**Keywords:** humidity sensor, fast response, hysteresis, nonporous nanoparticle, response time, recovery time

## Abstract

Fast-response humidity sensors using nanomaterials are attractive and have been intensively studied. Among the various nanomaterials, nonporous inorganic nanoparticles are suitable for use in humidity sensitive films for sensors. Here, we focus on a nonporous inorganic nanoparticle film and investigate a humidity sensor using the film. Hysteresis error and a dynamic response to a change of humidity are fundamental specifications of humidity sensors. A humidity sensor using a 50 nm silica nanoparticle film shows a hysteresis error of 2% at 85% RH and a response/recovery time of 2.8/2.3 s in 30% RH to 70% RH. We also summarize response/recovery times and hysteresis errors of state-of-the-art humidity sensors. As compared to those of commercial sensors and porous nanoparticle-based sensors evaluated using saturated salt solutions, the fabricated sensor shows a comparative hysteresis error and shorter response time.

## 1. Introduction

Humidity sensing has been of great importance in environmental monitoring, planting in factories, and human care [1,2]. Humidity sensors using electrolytes, organic polymers, and porous ceramics have been reported in early studies [3,4] and polymer-based humidity sensors have been commercialized recently. Detecting mechanisms of humidity in conventional sensors are based on the adsorption/desorption of water molecules inside a humidity-sensitive film. In the case of a commercial polymer-based sensor, a humidity-sensitive film typically has electrolytes which produce mobile ions using water molecules [5]. Thus, the electrical resistance of a film is dependent on the humidity in air. While this mechanism has been widely used, a dynamic response to a humidity change has not been studied well. Because the dynamic response is limited to the diffusion of water molecules inside/outside of a polymer film, the response/recovery time of commercial sensors is usually more than 5 s. Grange et al. reported a polymer-based capacitive humidity sensor with a 10 s response time and 2% accuracy due to humidity hysteresis [6].

Recently, there have been intensive studies to improve the dynamic response of humidity sensors. Borini et al. reported a pioneering work demonstrating the ultrafast response (≈30 ms) of humidity sensors using a graphene oxide film with a 15-nm thickness [7]. Recently, the number of publications reporting a fast/ultrafast response of humidity sensors has rapidly increased (see the trends in the Supplementary Materials). Most publications used nanomaterials as a component of humidity sensors. Without the assistance of a peripheral circuit, these nanomaterials intrinsically showed a fast response to a change in humidity.

Among the several kinds of nanomaterials, we focus on inorganic nanoparticles as a component of humidity sensitive films. Size-regulated inorganic nanoparticles are chemically stable in air and a thin film can be formed by solution processes using colloidal nanoparticles. In previous studies, porous inorganic nanoparticles were used for humidity sensors to increase sensitivity because the nanopores inside nanoparticles easily condense water molecules [8,9,10,11,12]. Nanoparticle-based humidity sensors using nonporous nanoparticles smaller than 10 nm have also been reported [13,14,15,16]. We expect that nonporous inorganic nanoparticles smaller than 10 nm are not appropriate because porous capillaries between nanoparticles capture water molecules and cause humidity hysteresis. Because the desorption situation is different from the adsorption one in nanopore structure due to capillary condensation, irreversible change occurs on adsorption of gas molecules. [17]. However, a detailed investigation of the response time and humidity hysteresis in nonporous nanoparticles has not yet been done.

In this study, we investigate the humidity hysteresis and response time of a nonporous inorganic nanoparticle-based humidity sensor. Nonporous silica nanoparticles with 50 nm and 200 nm diameters were used to form a humidity-sensitive film. For humidity sensing, we utilized surface conduction via water molecular layers on the nanoparticles. We evaluated the humidity hysteresis in ascending/descending humidity levels and response times relative to dynamic changes in humidity. A remote detection of respiratory air by the sensors was shown. We summarized the response/recovery time and hysteresis error of state-of-the-art humidity sensors.

## 2. Materials and Methods

We prepared gold interdigitated electrodes (line and spacing: 20 μm) on substrates as follows: gold with a titanium adhesive layer (100 and 10 nm) was sputtered (j-sputter, ULVAC) on a silicon wafer (thickness: 525 μm, resistivity 1 Ω cm to 100 Ω cm, oxide thickness: 1 μm). The gold/titanium layer was patterned into interdigitated structures with photolithography and dry-etching processes. The finished electrodes were electrically isolated under high humidity.

Colloidal silica nanoparticles were purchased from Sigma-Aldrich (product no.: “803073-1ML” and “803847-1ML”). The densities of the 50 and 200 nm nanoparticle colloids were diluted by adding ethanol 10 and 100 times, respectively. The colloids were spin-coated on bare interdigitated electrodes in ambient air. After the coating process, the substrate was heated to evaporate the solvent from the film.

Static humidity dependence of impedance was obtained in an environment-controlled chamber (SH-222, ESPEC, Osaka, Japan). The chamber was controlled at 34% to 90% relative humidity (RH) with 3% accuracy at 20 °C. The test pressure was 1 atm and the temperature variation was ±0.3 °C. The impedance was measured by an LCR meter (ZM2376, NF instruments, Kanagawa, Japan), which was controlled using LabVIEW (National Instruments, Austin, TX, USA). A voltage amplitude for impedance measurements was 1.0 V and a voltage frequency was 0.5 Hz to 1 kHz.

A dynamic response of impedance to a change of humidity was observed by using vessels with salt solutions. The volume of the vessels was 50 mL and the detailed dimensions are shown in the Supplementary Materials. K_2_CO_3_, Mg(NO_3_)_2_, NaBr, NaCl, KCl, and KNO_3_ salts were used in this study. Abrupt changes in humidity were given by the sensor which was placed in and removed from the vessel. The zero condition of the change is in ambient air without airflow. The temperature, relative humidity, and gas pressure are 20 °C, 30% RH, and 1 atm, respectively. The actual humidity in the vessels was simultaneously monitored by a commercial humidity sensor (CHS-UGS, TDK, Tokyo, Japan). The measured humidity levels above the salt solutions, using K_2_CO_3_, Mg(NO_3_)_2_, NaBr, NaCl, KCl, and KNO_3_, were 36, 46, 52, 61, 66, 70% RH, respectively. It should be noted that we only investigated the static response time without gas flow. The sampling period of the sensor impedance was 1 s and the applied voltage was 1 V at 1 Hz.

We also tested the availability of the nanoparticle-based sensor for the detection of respiration. Exhaled air has high moisture and temperature, is close to body temperature, and therefore, cyclically changed both the moisture and temperature of the air in front of the face. A mockup with a portable data logger (MetaWearC, MbientLab, San Francisco, CA, USA) was packaged in a plastic test case (width: 35 mm, height: 20 mm, depth: 50 mm; TW4-2-5G, Takachi Electronic Enclosure Co. Ltd, Saitama, Japan) and a tablet with a custom-made Android API (an intrinsic source code is available from MbientLab) was used. Interdigitated electrodes on a glass (line and spacing: 100 μm) were used for the substrate of the sensor. A 50 nm nanoparticle coating was carried out by using the same method as above. A change of resistive impedance was converted to a voltage by using a voltage divider comprised with 10 MΩ. Collected data were transmitted to the tablet via Bluetooth. The measurement was approved by the internal review board.

## 3. Results

### 3.1. Sensor Structure

**Figure 1:**
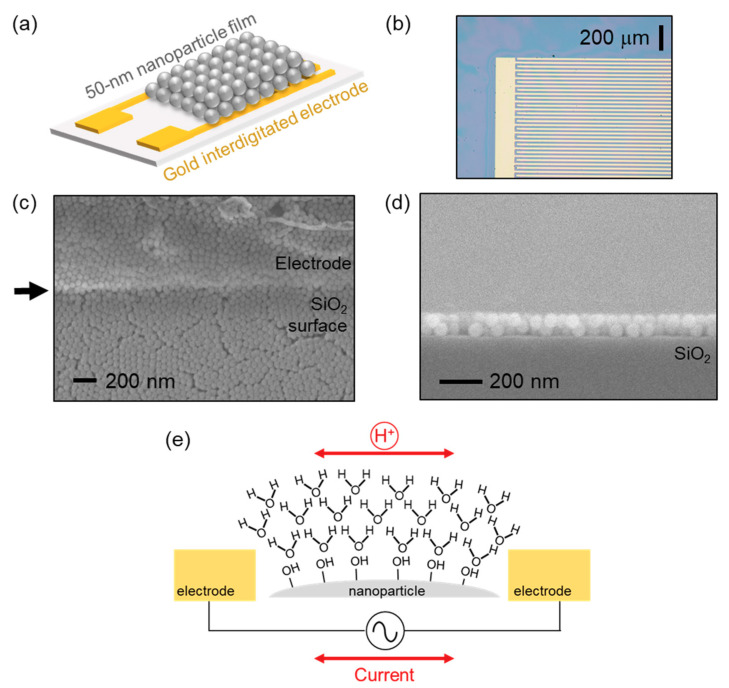
(**a**) Schematic of the device structure. (**b**) Optical microscope image of nanoparticle-coated electrodes. (**c**) Top-view and (**d**) cross-section SEM images of the device. (**e**) Schematic of proton conduction over nanoparticle surface in a moist environment.

Figure 1a shows a schematic of a humidity sensor using a 50 nm nanoparticle film and interdigitated electrodes. Figure 1b is an optical microscope image of nanoparticle-coated electrodes. Thin-film interference due to nanoparticles appears on the surface of an oxidized silicon. Detailed optical images of electrodes, before/after the coating process, are shown in the Supplementary Materials.

A morphology and thickness of nanoparticle films were observed by a field-emission scanning electron microscope (FE-SEM: S-4800, Hitachi High-Tech, Tokyo, Japan). Top-view and cross-section SEM images are shown in Figure 1c,d, respectively. Nanoparticles are in contact with neighboring particles. The film continuously covers over the surface as well as the edge of the electrode indicated by the arrow in Figure 1c. This guarantees that a proton conduction over a nanoparticle film occurs between electrodes. The film thickness in this image is approximately 100 nm on a SiO_2_ surface.

Figure 1e schematically describes a proton conduction over an oxide nanoparticle film in a moist environment. According to Seiyama et al., water molecules in air can have both chemisorption and physisorption on the surface of oxides [3]. In the case of chemisorption, water molecules are dissociated to form hydroxyl termination (-OH) with oxides on the utmost surface. This reaction is irreversible under room temperature because the activation energy of dissociation is much higher than the thermal energy (0.026 eV). Zhuravlev discussed that the activation energy of the desorption of hydroxyl monolayers from silica surface was 79 kJ/mol to 209 kJ/mol (0.8 eV to 2.2 eV per molecule) [18]. Therefore, chemisorption causes an irreversible humidity dependence in ascending/descending humidity levels.

On the contrary, water molecules physically form multilayers on surface hydroxyls via hydrogen bonds. This physisorption is reversible and the thickness of the multilayers is dependent on humidity in air. Using attenuated total reflection infrared (ATR-IR) spectroscopy, Chen et al. investigated the thickness of physically adsorbed water layers over hydroxyls on a silicon wafer [19]. They found that the water layers formed up to four layers from 0 to 90% RH. Therefore, the amount of physisorbed water molecules is dependent on surrounding humidity. As more than two water layers form, protons can hop through a chain of water molecules via hydrogen bonds [20]. According to Agmon, the activation energy of proton mobility was 8.3 kJ/mol to 12.5 kJ/mol (0.087 eV to 0.13 eV per molecule). In this work, this energy is given by an AC electric field and the surface conduction results in humidity dependence of the impedance. It should be noted that a proton conduction attributes to a reduction of a resistive impedance in the film.

### 3.2. Sensor Characteristics: Hysteresis Error

**Figure 2:**
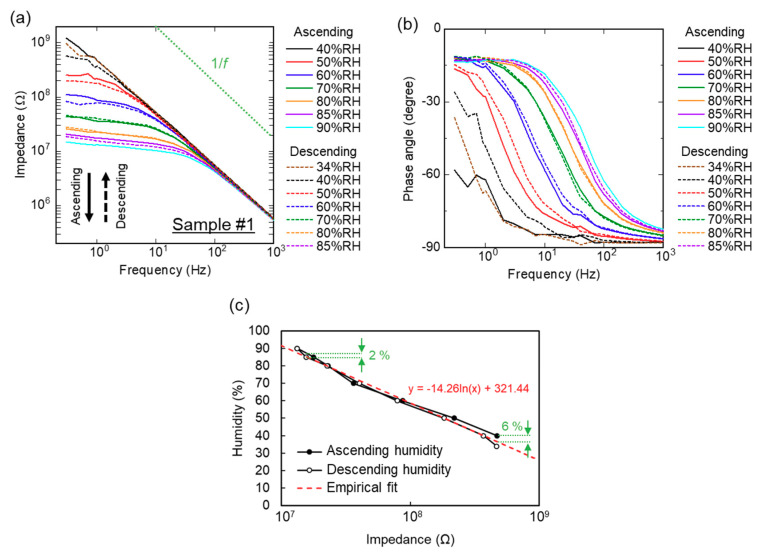
Impedance analysis of a 50 nm nanoparticle film. (**a**,**b**) Frequency dependence. Solid and dashed lines are in an ascending and descending humidity level, respectively. (**a**) Impedance and (**b**) phase angle of impedance. (**c**) Humidity dependence of impedance at *f* = 1 Hz. A dashed line is an empirical fit using a logarithmic function.

Figure 2a,b shows a frequency (*f*) dependence of impedance in a 50 nm nanoparticle film as a function of humidity (a): absolute value and (b) phase angle of impedance). Solid and dashed lines represent the measurements in the ascending and descending humidity levels, respectively. As a humidity increases from 40% RH to 90% RH, the impedance at a lower frequency decreases and shows a plateau region. This plateau region is attributed to a resistive impedance of proton conduction (i.e., a phase angle of impedance is slightly lower than zero degrees). A linear decrease in impedance (*Z*), which is independent on humidity, is due to a parasitic capacitance (*C_p_*) of the measurement system: i.e., |*Z*|∝(*ωC_p_*)^−1^ = (2π*fC*_p_)^−1^ where |*Z*| is an absolute value of impedance and *ω* is an angular frequency (ω = 2π*f*) of an AC voltage.

Figure 2c plots a relation of film impedance and humidity. The fixed frequency was 1 Hz to evaluate resistive impedance in this study. The impedance changed by one order of magnitude from 34% RH to 90% RH. We fit an empirical logarithmic function to the plots as H=−14.26ln(Z)+321.44, where *H* is a relative humidity in percentage. In other words, we express this relation as $Z\propto \exp\left(-\frac{H}{14.26}\right)$. For practical usage, it is important to give a mathematical equation to show the relation between impedance and relative humidity. By using empirical fitting, the impedance can be converted to relative humidity in air.

This exponential relation can be attributed to the formation of water layers. According to Asay et al., a structural evolution of water molecular formation has three steps on a flat silicon-oxide surface [21]. First, an ice-like water monolayer grows from 0% RH to 30% RH, which does not contribute to proton conduction. Second, from 30% RH to 60% RH, approximately one molecular layer continues to grow over the ice-like water. This causes proton conduction through the water layers to start. Third, over 60% RH, liquid-like water layers form on the surface and this bulk water enhances proton conduction and decreases impedance. Seo et al. discussed the relation between the evolution of water and a proton conduction over a thermally oxidized silicon surface [22]. They observed an exponential increase of current versus humidity (≈0 nA at <60% RH and 12 nA at 80% RH in DC current): current conduction was enhanced by liquid-like water.

**Figure 3:**
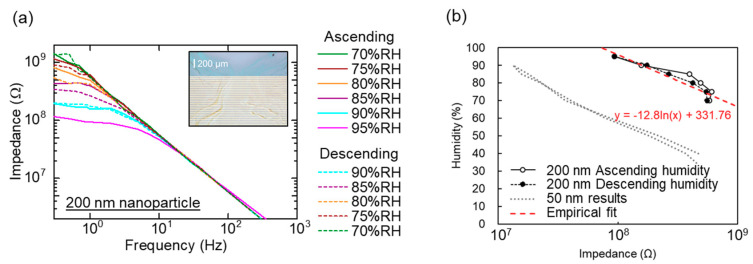
Impedance analysis of a 200 nm nanoparticle film. (**a**) Frequency dependence as a function of humidity. Processes of ascending and descending humidity levels are described as solid and dashed lines, respectively. Inset: nanoparticle-coated electrodes. (**b**) Humidity dependence of impedance of a 200 nm nanoparticle film at 1 Hz. Dotted lines are the ascending/descending humidity level of a 50 nm nanoparticle film (Figure 2c).

The hysteresis error is calculated by the difference between the experimental results and the empirical fitting line. We evaluated maximum humidity hysteresis at 6% at 40% RH and 2% at 85% RH in the ascending/descending humidity levels. At a lower humidity, the impedance becomes larger and its accuracy is reduced. This nanoparticle humidity sensor based on proton conduction is suitable for detecting higher humidities. It should be noted that a humidity sensor using 200 nm silica nanoparticles shows 10 times larger impedance although an exponential dependence appears (Figure 3a,b). Because it is easier to introduce a lower impedance element into electrical circuits, 50 nm nanoparticles are more suitable for nanoparticle films to sense humidity.

### 3.3. Sensor Characteristics: Response/Recovery Time

**Figure 4:**
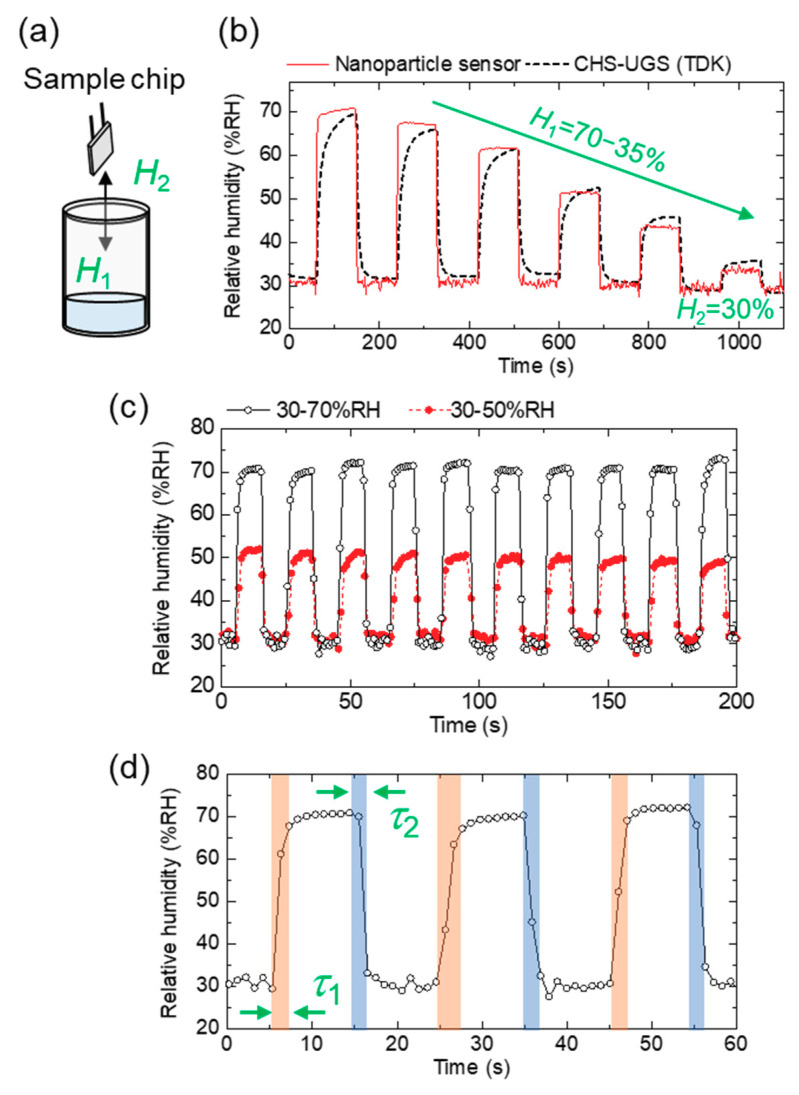
(**a**) Schematic of a measurement setup for dynamic response to a humidity change. (**b**) Dynamic response of a nanoparticle sensor (solid line) and a commercial sensor (CHS-UGS, dash line, TDK Corporation, Tokyo, Japan). (**c**) Repetitive dynamic response of a sensor to a humidity change. (**d**) Evaluation of response and recovery time.

A schematic setup for measuring a dynamic response to a humidity change is shown in Figure 4a. Both a fabricated sensor chip and a commercial humidity sensor are introduced in and taken out from the vessels. Here, *H*_1_ and *H*_2_ represent as relative humidity in and out of the vessel, respectively. Figure 4b shows a dynamic response of the both sensors exposed to a humidity change simultaneously. We found that the nanoparticle-based sensor had a shorter response/recovery time than the commercial sensor. Figure 4c shows a repetitive dynamic response to a humidity change (30% RH to 50% RH and 30% RH to 70% RH). Response time (*τ*_1_) and recovery time (*τ*_2_) in 30% RH to 70% RH are evaluated to be 2.8 and 2.3 s, which are defined as *t*_90_ and *t*_10_ (a time required for a 90% of the total change in impedance) are shown in Figure 4d. Time *t*_90_ and *t*_10_ were evaluated using a graphical readout. We assumed that the uncertainty of the measured response times was up to 1 s because the sampling period was approximately 1 s.

**Figure 5:**
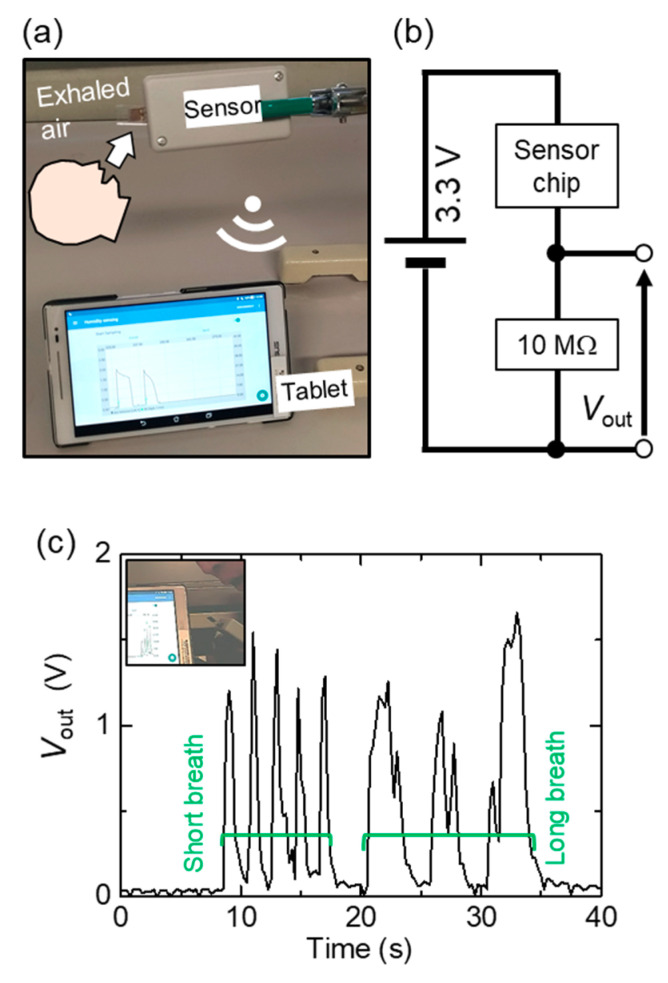
Demonstration of remote sensing of respiration. (**a**) Picture of the setup, (**b**) circuit to convert impedance to output voltage (*V*_out_), and (**c**) response to respiration. Inset in (**c**) is the picture of exhaled air given to the sensor.

Using this fast response/recovery time, we show a proof-of-concept test for practical applications of a nanoparticle humidity sensor. Because a common respiratory rate ranges from 12 bpm to 20 bpm (0.05 Hz to 0.083 Hz), a humidity sensor with a 2.8/2.3 s response/recovery time can be used for sensing respiratory rate. A setup of remote sensing of respiration is shown in Figure 5a. A voltage change due to an impedance change is remotely collected in a tablet via Bluetooth. Breath air changes both moisture and temperature of the air in front of the face. In this proof-of-concept mockup, the sensor does not measure relative humidity of exhaled air but only finds a large amount of water vapor qualitatively. Figure 5b is the electrical circuit to convert a change of impedance to output voltage (*V*_out_). Expiratory air is successively detected in 2 s with sharp voltage signals, as shown in Figure 5c, which guarantees the possible application of the humidity sensor for respiratory-rate detection. In the Supplementary Materials, another example of breath detection is shown. In this device mockup, we cannot convert *V*_out_ to actual relative humidity because temperature sensing is required for the conversion. In this proof-of-concept, the sensor does not measure relative humidity of exhaled air but only detects the existence of water vapor qualitatively. The measurement of relative humidity in breath air is a cutting-edge topic and further developments are required in our system [23].

## 4. Discussions

### 4.1. Intrinsic and Extrinsic Response Time

It should be noted that there are two types of response times regarding diffusion of water molecules: dominated by intrinsic (sensor related) and extrinsic (environment related) processes [24]. Sensor response/recovery time (*τ*) is equivalent to the total of the mixing time to obtain certain humidity in the chamber (*τ_m_*), the adsorption time of water molecules onto the sensor (*τ_a_*), and the time required for the change of the sensor property (*τ_c_*): *τ* = *τ_m_* + *τ_a_* + *τ_c_*. The value of *τ_m_* is related to extrinsic response and *τ_a_* and *τ_c_* are attributed to intrinsic response.

In the case of the recovery process, because the volume out of the vessel (ambient air) is much larger than that of the moist air in the vessel (50 mL), *τ_m_* is reduced and the evaluated recovery time is dominated by the intrinsic *τ_a_* and *τ_c_*. On the other hand, the evaluated response time may include the effect of *τ_m_* because the response time (2.8 s) is longer than the recovery time (2.3 s), as shown in Figure 4d.

### 4.2. Specification with State-of-the-Art Humidity Sensors

**Figure 6:**
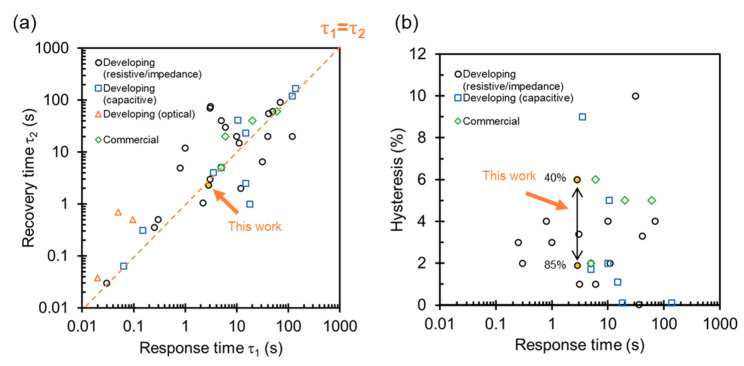
Specifications with state-of-the-art humidity sensors using nanomaterials. (**a**) Response time versus recovery time. (**b**) Response time versus hysteresis error. We classify the plots by sensing techniques: developing and commercial sensors. A list of literature is shown in the Supplementary Materials.

We finally discuss sensor specifications, a response/recovery time, and a humidity hysteresis with state-of-the-art humidity sensors using nanomaterials. Figure 6a,b show the overall survey of a response/recovery time and a humidity hysteresis. The list of literature is shown in the Supplementary Materials. We also plot specifications of commercial humidity sensors as rhombus marks. In the literature, two measurements of response/recovery time are used—using a humidity-controlled chamber and a vessel with a saturated salt solution. By using a humidity-controlled chamber, an extrinsic *τ_m_* to obtain certain humidity is included in the evaluation of response/recovery time. In case of using a saturated salt solution, the effect of extrinsic *τ_m_* can be reduced because the humidity is stable at certain values in the vessel [25]. Therefore, response/recovery time measured by a humidity-controlled chamber tends to be longer than that measured by using saturated salt solutions. As a guide for the eye, the dashed line in (a) represents the boundary of the response and recovery time are identical.

Table 1 summarizes specifications of recently reported nanoparticle-based humidity sensors. Our result using a nonporous nanoparticle film shows a comparable response/recovery time with the sensors using porous nanoparticles evaluated by a saturated salt solution. The faster recovery time indicates that the amount of trapped water is small enough to keep a quick response due to the nanoparticle surface. Recently, humidity sensors with an ultrafast response (<1 s) [7,10,26,27,28,29] and a small hysteresis error (<1%) [11,30,31] have been reported. In the future, we will aim at further improvements of the specifications of nonporous inorganic nanoparticle-based sensors.

It should be noted that the specifications in Figure 6 and Table 1 were obtained by measuring bare sensor chips. Implementation of peripheral interface circuits can enhance the specifications of humidity sensitive chips. For example, linearity of output to humidity is critical for practical usage. Therefore, a differentiation circuit was proposed to provide a linear output of humidity from an exponential change of impedance [32]. Noise-resistance ability of sensors can be improved by using peripheral circuits such as digital/analog filters. Toward implementation into RFID tags, a CMOS-compatible humidity sensor was proposed to have a high sensitivity of 1.4 fF/% RH with noise suppression by electrical circuits [33]. Environmental conditions, such as temperature, influence sensing performance. Although the reported sensor in this paper does not have a temperature sensor, simultaneous monitoring of temperature is an essential technique to calibrate the influence digitally.

### 4.3. Repeatability

In order to guarantee practical usability, it is essential to achieve repeatability of sensors. Therefore, we show the humidity dependence of impedance on other two sensor chips in the Supplementary Materials. Large reduction of impedance is also observed as the humidity increases; however, the humidity dependence of impedance is not identical as Figure 4c The values of slope in the empirical fitting are −13.11 and −13.06 in the semi-logarithmic plot while that in Figure 4c is −14.26. This variation is possibly attributed to the geometrical variation of nanoparticle films between electrodes. It is inevitable to have a less-ordered structure in nanoparticle films by spin-coating. More sophisticated techniques are feasible to assemble colloidal nanoparticles into highly ordered films, such as ink-jet printing and micromolding methods [36]. These methods can produce a confined 3D structure with nanoparticles in desired positions.

## 5. Conclusions

In summary, we investigated a humidity hysteresis and a response time of a nonporous inorganic nanoparticle-based humidity sensor. A nonporous nanoparticle film was potential to enhance fast humidity sensing with small hysteresis. A humidity hysteresis and a response/recovery time of a 50 nm nanoparticle film were evaluated to be as 2% to 6% (at 85% RH to 40% RH) and 2.8/2.3 s (a change of humidity between 30% RH to 70% RH), respectively. We demonstrated a remote sensing of respiration. According to the specifications with state-of-the-art sensors, the nonporous nanoparticle sensor showed a faster response than commercial humidity sensors and porous nanoparticle-based sensors evaluated by using saturated salt solutions. Repeatability of the sensors should be improved in future works towards practical usage.

## Figures and Tables

**Table 1:** Response/recovery time and hysteresis error in porous/nonporous nanoparticle-based humidity sensors.

Materials	Porous/Nonporous Particles	Particle Diameter (nm)	Response Time/Recovery Time (s)	Method (Chamber/Salt)	Hysteresis (%)	Reference
SnS_2_ nanoflower/Zn_2_SoO_4_ hollow sphere	Porous	400	18/2	Salt	0.1	[30]
Porous silica nanoparticle aerogel	Porous	30	41/55	Chamber	3.3	[8]
Microporous silica nanoparticle	Porous	105	5/40	Salt	2	[9]
Porous silicon nanoparticle	Porous	900	36/NA	Chamber	0.02	[11]
TiO_2_ nanoparticles/polypyrrole composite	Nonporous	7	40/20	Chamber	NA	[34]
La_0.7_Sr_0.3_MnO_3_ nanocrystal	Nonporous	20 to 40	0.8/4.9	Salt	4	[35]
In_2_O_3_ nanocube/graphene oxide nanosheet	Nonporous	20 to 40	15/2.5	Salt	NA	[2]
Silica nanoparticle	Nonporous	50	2.8/2.3	Salt	2–6	This work
HTU21 (TE connectivity, commercial sensor)			5/5 *	Chamber	2	Datasheet

NA: not shown, * evaluated by using *t*_63_.

## Supplementary Materials

The following are available online at https://www.mdpi.com/1424-8220/20/14/3858/s1, Figure S1: Numbers of publications reporting fast-response humidity sensors, Figure S2: Dimensions of the glass vessel used for the experiments, Figure S3: Optical microscope images of nanoparticle-coated electrodes, Figure S4: Another example of breath detection, Figure S5: Humidity dependence of impedance on other sensors, Table S1: List of literature for comparison of specifications (response time and recovery time), Table S2: List of literature for comparison of specifications (response time and humidity hysteresis), Table S3: List of commercial humidity sensors.
