# Supplementary material for: Nonporous Inorganic Nanoparticle-Based Humidity Sensor: Evaluation of Humidity Hysteresis and Response Time
Source: Sensors (Basel). 2020 Jul 10;20(14):3858. doi: 10.3390/s20143858 (PMC7411755; doi:10.3390/s20143858)
Supplement: Supplementary file 1 [file sensors-20-03858-s001.pdf]

Supplementary Materials

Nonporous inorganic nanoparticle-based humidity sensor: evaluation of humidity hysteresis and response time

Shinya Kano [a)] and Harutaka Mekaru

Sensing System Research Center, National Institute of Advanced Industrial Science and Technology (AIST), Tsukuba, 305-8564, Japan

[a)] Author to whom correspondence should be addressed: shinya-kano@aist.go.jp

FIG. S1 Numbers of publications reporting fast-response humidity sensors in each year. 2
FIG. S2 Dimensions of the glass vessel (volume: 50 ml) used for the experiments. 3
FIG. S3 Optical microscope images of nanoparticle-coated electrodes. 4
FIG. S4 Another example of breath detection. 5
FIG. S5 Humidity dependence of impedance on other sensors (#2 and #3). 6
Table S1 List of literature for comparison of specifications (response time and recovery time). 7
Table S2 List of literature for comparison of specifications (response time and humidity hysteresis). 9
Table S3 List of commercial humidity sensors. 10

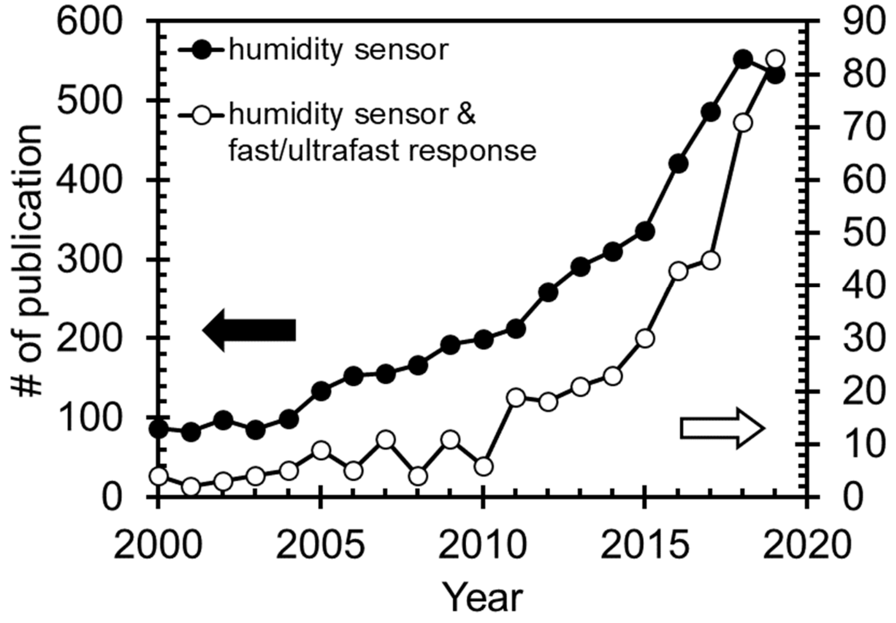

FIG. S1 Numbers of publications reporting fast-response humidity sensors in each year.
(survey date: 26th May 2020, Web of Knowledge)
Keywords of "humidity sensor/sensors, fast response, and ultrafast" are used for the survey.

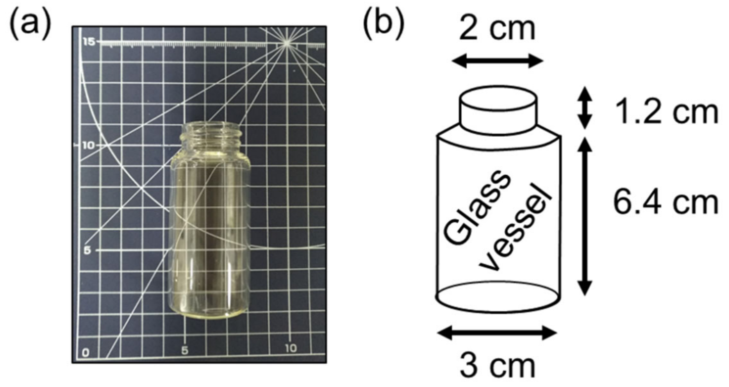

FIG. S2 Dimensions of the glass vessel (volume: 50 ml) used for the experiments. (a) Photo and (b) detailed dimensions are shown.

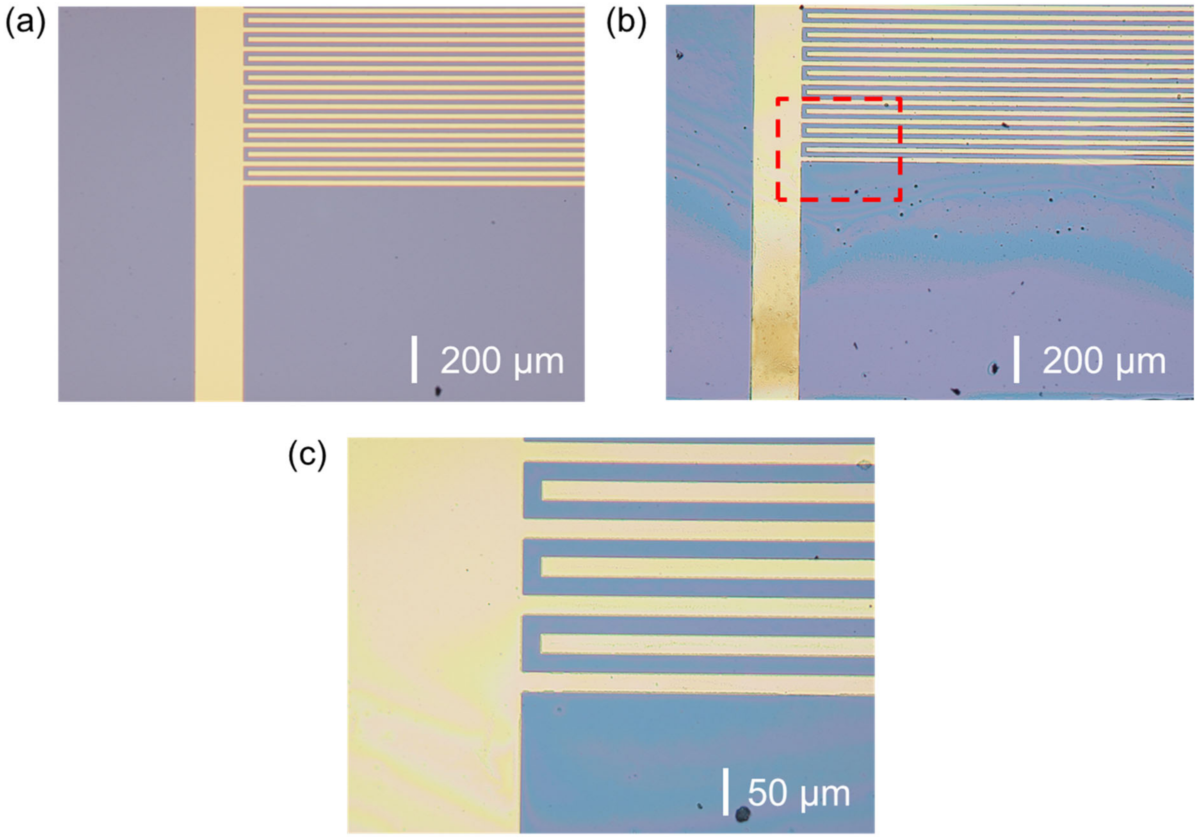

FIG. S3 Optical microscope images of nanoparticle-coated electrodes.
(a) Before and (b) after the coating process. (c) Enlarged area in (b) indicated by the dashed square.

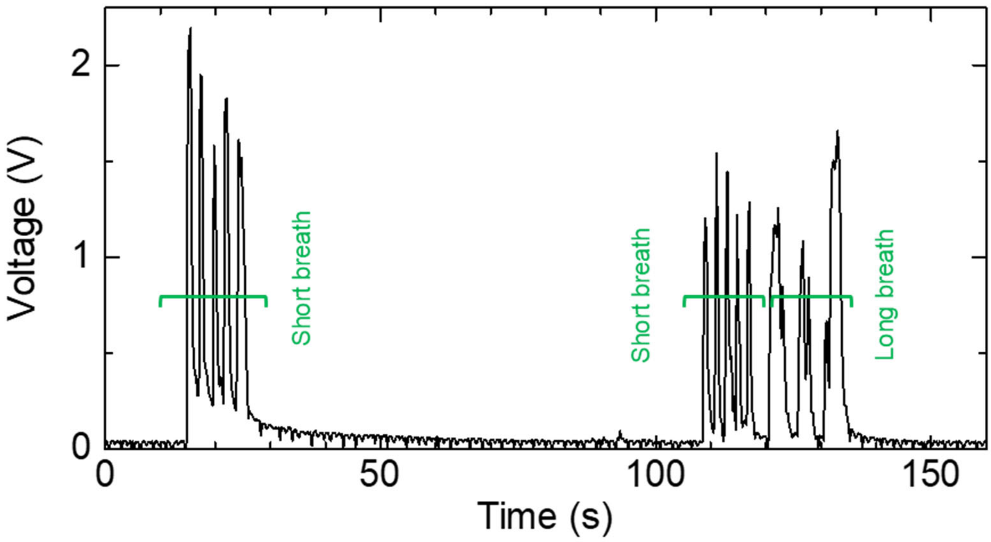

FIG. S4 Another example of breath detection.

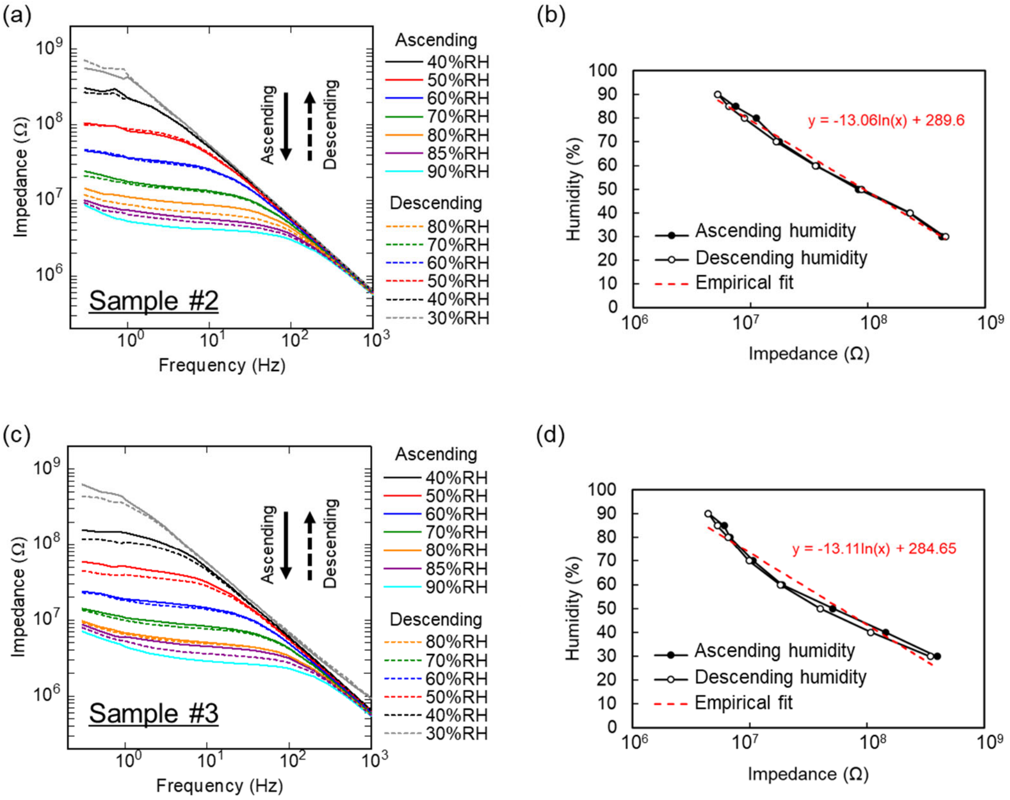

FIG. S5 Humidity dependence of impedance on other sensors (#2 and #3).
(a) and (c): Frequency dependence. (b) and (d): humidity versus impedance plot at 1 Hz. A dashed line in (b) and (d) is an empirical fit using a logarithmic function.

Table S1 List of literature for comparison of specifications (response time and recovery time).

Materials	Type	Response time (s)	Recovery time (s)	Humidity change (%)	Reference
Nanostructured TiO_2	capacitive	0.064	0.064	2-95	J. Steele, Sens. Act. B 2009[1]
HMDS-modified silicon nanowire	capacitive	15	23	11.3-57	X. Chen, Sens.Act B 2011[2]
Graphene oxide	capacitive	10.5	41	23-86	H. Bi, Sci. Rep. 2013[3]
Polyimide with carbon nanotube electrode	capacitive	0.15	0.31	0-100	E. Itoh, JJAP 2017[4]
SnS_2 nanoflower/Zn_2SoO_4 hollow sphere	capacitive	18	1	0-97	D. Zhang ACS AMI 2018[5]
ZnO/MoS_2	capacitive	138	166	25-85	D. Burman, IEEE Sens. 2019[6]
Polyester fiber	capacitive	3.5	4	6-33	L. Ma, Adv. Func. Mat. 2019[7]
In_2O_3 nanocube/graphene oxide nanosheet	capacitive	15	2.5	0-43	B. Li, Sens. Act. B 2019[8]
PDMS-$CaCl_2$	capacitive	120	120	30-60	Y. Komazaki, Sens. Act. B 2019[9]
$CsPbBr_3/TiO_2$	capacitive	5	5	40-88	M.-Y. Cho, Adv. Func. Mat. 2020[10]
Porous silica nanoparticle aerogel	impedance	41	55	20-80	C. Wang, Sens. Act. B 2005[11]
TiO_2 nanoparticles/polypyrrole composite	impedance	40	20	30-80	P. Su, Sens. Act. B 2007[12]
Gallium-doped ZnO	impedance	70	90	30-90	P. Su, Sens. Act. B 2009[13]
Al_2O_3 nanotube film	impedance	10	20	11-95	B. Cheng, J. Mater. Chem. 2011[14]
Graphene oxide	impedance	0.03	0.03	—	S. Borini, ACS Sens. 2013[15]
Graphene/methyl-red	impedance	0.251	0.35	35-100	S. Ali, Carbon 2016[16]

Poly(ionic liquid)s	impedance	6	30	11-95	L. Wang, Chem. Comm. 2016[17]
$TiO_2/SrTiO_3$ composite	impedance	3.1	76	11-75	M. Zhang, Sensors 2017[18]
$La_{0.7}Sr_{0.3}MnO_3$ nanocrystal film	impedance	0.8	4.9	11-95	Z. Duan, Sens. Act. B 2018[19]
<10 nm silica nanoparticle	impedance	31.4	6.5	30-84	S. Kano, ACS Sustain. Chem. Eng. 2018[20]
Microporous silica nanoparticle	impedance	5	40	11-95	H. Zhao, Sens. Act. B 2018[21]
Graphite	impedance	1	12	11-95	Y. Zhang, Sens. Act. B 2018[22]
$TiO_2/NaNbO_3$	impedance	11	15	12-94	R. Si, Sens. Act. B 2020[23]
Attapulgite	impedance	3	70	0-91.5	Z. Duan, Sens. Act. B 2020[24]
50-nm silica nanoparticle	impedance	2	2	30-70	This work
SnO single nanowire	resistive	120	20	5-85	Q. Kuang, J. Am. Chem. Soc. 2007[25]
CeO_2 nanowires	resistive	3	3	15.2-85	X. Fu, Nanotechnology 2007[26]
$NiMn_2O_3$ nanoparticle	resistive	50	60	11-92	Y. Gawli, Sens. Act. B 2014[27]
Supramolecular nanofibers	resistive	2.2	1.05	5-65	U. Mogera, Sci. Rep. 2014[28]
Silicon nanocrystal film	resistive	12	2	20-95	S. Kano, ACS Sens. 2017[29]
MoO_3 nanosheet	resistive	0.3	0.5	0-40	J. Yang, Small 2019[30]
Agarose coated fiber	optical	0.05	0.7	60-90	J. Mathew, Sens. Act. A 2012[31]
Graphene oxide	optical	0.096	0.5	—	X. Gan, Appl. Phys. Lett. 2017[32]
Titania microsphere	optical	0.02	0.038	20-90	S. Mohd-Noor J. Mater. Chem. A 2019[33]

Table S2 List of literature for comparison of specifications (response time and humidity hysteresis).

Materials	Type	Response time (s)	hysteresis (%)	Reference
Polymer	Capacitive	10	2	H. Grange, Sens. Act. 1987[34]
HMDS-modified silicon nanowire	Capacitive	15	1.1	X. Chen, Sens. Act B 2011[2]
Graphene oxide	Capacitive	10.5	5	H. Bi, Sci. Rep. 2013[3]
SnS_2 nanoflower/Zn_2SoO_4 hollow sphere	Capacitive	18	0.1	D. Zhang ACS AMI 2018[5]
ZnO/MoS_2	capacitive	138	0.1	D. Burman, IEEE Sens. 2019[6]
Polyester fiber	capacitive	3.5	9	L. Ma, Adv. Func. Mat. 2019[7]
$CsPbBr_3/TiO_2$	capacitive	5	1.7	M.-Y. Cho, Adv. Func. Mat. 2020[10]
Porous silicon	capacitance	90	6	M. Bjorkqvist, Sens. Act. A 2004[35]
Porous silica nanoparticle aerogel	impedance	41	3.3	C. Wang, Sens. Act. B 2005[36]
Gallium-doped ZnO	impedance	70	4	P. Su, Sens. Act. B 2009[13]
Al_2O_3 nanotube film	impedance	10	4	B. Cheng, J. Mater. Chem. 2011[14]
Graphene/methyl-red	impedance	0.251	3	S. Ali, Carbon 2016[16]
Poly(ionic liquid)s	impedance	6	1	L. Wang, Chem. Comm. 2016[17]
$TiO_2/SrTiO_3$ composite	impedance	3.1	1	M. Zhang, Sensors 2017[18]
$La_{0.7}Sr_{0.3}MnO_3$ nanocrystal film	impedance	0.8	4	Z. Duan, Sens. Act. B 2018[19]
<10 nm silica nanoparticle	impedance	31.4	10	S. Kano, ACS Sustain. Chem. Eng. 2018[20]
Microporous silica nanoparticle	impedance	5	2	H. Zhao, Sens. Act. B 2018[21]
Graphite	impedance	1	3	Y. Zhang, Sens. Act. B 2018[22]
$TiO_2/NaNbO_3$	impedance	11	2	R. Si, Sens. Act. B 2020[23]
Attapulgite	impedance	3	3.4	Z. Duan, Sens. Act. B 2020[24]
50-nm silica nanoparticle film	impedance	2	5	This work
MoO_3 nanosheet	resistive	0.3	2	J. Yang, Small 2019[30]
Porous silicon nanoparticles	resistive	36	0.02	T. Jalkanen, Appl. Phys. Lett. 2012[37]

Table S3 List of commercial humidity sensors.

Sensor (manufacture)	Type	Response time (s)	Recovery time (s)	A change of humidity (%)	Hysteresis error (%)
HIH-4000 (Honeywell)	capacitive	6	20	—	6
CHS-UGS (TDK)	impedance	60	60	30-85%	5
HR202L (Aosong Guangzhou Electronics)	impedance	20	40	—	5
HTU21 (TE connectivity)	capacitive	5	5	33-75%	2

Reference

[1] J.J. Steele, M.T. Taschuk, and M.J. Brett, Sensors Actuators, B Chem. **140**, 610 (2009).

[2] X. Chen, J. Zhang, Z. Wang, Q. Yan, and S. Hui, Sensors Actuators, B Chem. **156**, 631 (2011).

[3] H. Bi, K. Yin, X. Xie, J. Ji, S. Wan, L. Sun, M. Terrones, and M.S. Dresselhaus, Sci. Rep. **3**, 2714 (2013).

[4] E. Itoh and Z. Yuan, Jpn. J. Appl. Phys. **56**, 05EC03 (2017).

[5] D. Zhang, X. Zong, Z. Wu, and Y. Zhang, ACS Appl. Mater. Interfaces **10**, 32631 (2018).

[6] D. Burman, D.S. Choudhary, and P.K. Guha, IEEE Sens. J. **19**, 3993 (2019).

[7] L. Ma, R. Wu, A. Patil, S. Zhu, Z. Meng, H. Meng, C. Hou, Y. Zhang, Q. Liu, R. Yu, J. Wang, N. Lin, and X.Y. Liu, Adv. Funct. Mater. **29**, 1 (2019).

[8] B. Li, Q. Tian, H. Su, X. Wang, T. Wang, and D. Zhang, Sensors Actuators, B Chem. **299**, 126973 (2019).

[9] Y. Komazaki and S. Uemura, Sensors Actuators B Chem. **297**, 126711 (2019).

[10] M.Y. Cho, S. Kim, I.S. Kim, E.S. Kim, Z.J. Wang, N.Y. Kim, S.W. Kim, and J.M. Oh, Adv. Funct. Mater. **30**, 1 (2020).

[11] H. Wang, S. Chen, L. Li, and S. Jiang, **21**, 2003 (2005).

[12] P.G. Su and L.N. Huang, Sensors Actuators, B Chem. **123**, 501 (2007).

[13] P.G. Su, J.Y. Tseng, Y.C. Huang, H.H. Pan, and P.C. Li, Sensors Actuators, B Chem. **137**, 496 (2009).

[14] B. Cheng, B. Tian, C. Xie, Y. Xiao, and S. Lei, J. Mater. Chem. **21**, 1907 (2011).

[15] S. Borini, R. White, D. Wei, M. Astley, S. Haque, E. Spigone, N. Harris, J. Kivioja, and T. Ryhänen, ACS Nano **7**, 11166 (2013).

[16] S. Ali, A. Hassan, G. Hassan, J. Bae, and C.H. Lee, Carbon N. Y. **105**, 23 (2016).

[17] L. Wang, X. Duan, W. Xie, Q. Li, and T. Wang, Chem. Commun. **52**, 8417 (2016).

[18] M. Zhang, S. Wei, W. Ren, and R. Wu, Sensors **17**, 1310 (2017).

[19] Z. Duan, M. Xu, T. Li, Y. Zhang, and H. Zou, Sensors Actuators, B Chem. **258**, 527 (2018).

[20] S. Kano and M. Fujii, ACS Sustain. Chem. Eng. **6**, 12217 (2018).

[21] H. Zhao, T. Zhang, R. Qi, J. Dai, S. Liu, T. Fei, and G. Lu, Sensors Actuators, B Chem. **266**, 131 (2018).

[22] Y. Zhang, Z. Duan, H. Zou, and M. Ma, Sensors Actuators, B Chem. **261**, 345 (2018).

[23] R. Si, X. Xie, T. Li, J. Zheng, C. Cheng, and C. Wang, Sensors Actuators, B Chem. **309**, (2020).

[24] Z. Duan, Q. Zhao, S. Wang, Z. Yuan, Y. Zhang, X. Li, Y. Wu, Y. Jiang, and H. Tai, Sensors Actuators, B Chem. **305**, 127534 (2020).

[25] Q. Kuang, C. Lao, Z.L. Wang, Z. Xie, and L. Zheng, J. Am. Chem. Soc. **129**, 6070 (2007).

[26] X.Q. Fu, C. Wang, H.C. Yu, Y.G. Wang, and T.H. Wang, Nanotechnology **18**, 145503 (2007).

[27] Y. Gawli, S. Badadhe, A. Basu, D. Guin, M. V. Shelke, and S. Ogale, Sensors Actuators, B Chem. **191**, 837 (2014).

[28] U. Mogera, A.A. Sagade, S.J. George, and G.U. Kulkarni, Sci. Rep. **4**, 4103 (2014).

[29] S. Kano, K. Kim, and M. Fujii, ACS Sensors **2**, 828 (2017).

[30] J. Yang, R. Shi, Z. Lou, R. Chai, K. Jiang, and G. Shen, Small **15**, 1902801 (2019).

[31] J. Mathew, Y. Semenova, and G. Farrell, Sensors Actuators, A Phys. **174**, 47 (2012).

[32] X. Gan, C. Zhao, Q. Yuan, L. Fang, Y. Li, J. Yin, X. Ma, and J. Zhao, Appl. Phys. Lett. **110**, (2017).

[33] S. Mohd-Noor, H. Jang, K. Baek, Y.R. Pei, A.M. Alam, Y.H. Kim, I.S. Kim, J.H. Choy, and J.K. Hyun, J. Mater. Chem. A **7**, 10561 (2019).

[34] H. Grange, C. Bieth, H. Boucher, and G. Delapiere, Sensors and Actuators **12**, 291 (1987).

[35] M. Björkqvist, J. Salonen, J. Paski, and E. Laine, Sensors Actuators, A Phys. **112**, 244 (2004).

[36] C.-T. Wang, C.-L. Wu, I.-C. Chen, and Y.-H. Huang, Sensors Actuators B Chem. **107**, 402 (2005).

[37] T. Jalkanen, E. Mäkilä, A. Määttänen, J. Tuura, M. Kaasalainen, V.P. Lehto, P. Ihalainen, J. Peltonen, and J. Salonen, Appl. Phys. Lett. **101**, 263110 (2012).
